# Preoperative network activity predicts the response to subthalamic DBS for Parkinson’s disease

**DOI:** 10.21203/rs.3.rs-4178280/v2

**Published:** 2025-05-14

**Authors:** Prashin Unadkat, An Vo, Yilong Ma, Chris C. Tang, Vijay Dhawan, Martin Niethammer, Nha Nguyen, Shichun Peng, Akash Mishra, Ritesh Ramdhani, Albert Fenoy, Silvia Paola Caminiti, Daniela Perani, David Eidelberg

**Affiliations:** 1 Center for Neurosciences, The Feinstein Institutes for Medical Research, Manhasset, NY, USA; 2 Elmezzi Graduate School of Molecular Medicine, Manhasset, NY, USA; 3 Department of Neurosurgery, Donald and Barbara Zucker School of Medicine at Hofstra/Northwell, Hempstead, NY, USA; 4 Molecular Medicine, Donald and Barbara Zucker School of Medicine at Hofstra/Northwell, Hempstead, NY, USA; 5 Department of Neurology, Donald and Barbara Zucker School of Medicine at Hofstra/Northwell, Hempstead, NY, USA; 6 Institute of Behavioral Science, The Feinstein Institutes for Medical Research, Manhasset, NY, USA; 7 Department of Brain and Behavioral Sciences, University of Pavia, Pavia, Italy; 8 Vita-Salute San Raffaele University, Milan, Italy; 9 Nuclear Medicine Unit, San Raffaele Hospital, Milan, Italy

**Keywords:** deep brain stimulation (DBS), Parkinson’s disease (PD), subthalamic nucleus (STN), functional brain imaging, brain networks, biomarkers

## Abstract

Quantitative imaging markers to aid in the selection of Parkinson’s disease (PD) patients for surgical interventions such as subthalamic nucleus deep brain stimulation (STN-DBS) are currently lacking. Using metabolic PET and network analysis we identified and validated a treatment-induced topography, termed STN StimNet. Stimulation-mediated changes in network expression correlated with concurrent motor improvement in independent STN-DBS cohorts scanned on and off stimulation. Moreover, STN StimNet measurements off stimulation correlated with local field potentials recorded from the STN, whereas intraoperative modulation of cortical activity by STN stimulation correlated with contributions to the network from corresponding brain regions. These findings suggested that stimulation-mediated clinical responses are influenced by baseline StimNet expression. Indeed, we found that motor outcomes following STN-DBS were predicted by preoperative network expression measured using metabolic PET or resting state fMRI. To illustrate the potential utility of these measures in selecting optimal candidates for DBS surgery, STN StimNet expression was computed in scans from 175 PD patients (0–21 years from diagnosis). The resulting values were used to identify those individuals likely to derive meaningful benefit from a potential STN-DBS procedure. This approach suggests that preoperative network quantification provides unique information regarding baseline brain circuitry, which may be useful in surgical decision making.

## Introduction

Over three decades since the first reports of its therapeutic utility, deep brain stimulation (DBS) applied to the dorsolateral subthalamic nucleus (STN) has been consistently shown to improve motor symptoms in Parkinson’s disease (PD) patients, while reducing daily levodopa requirements and peak-dose dyskinesias [1–4]. Together, the benefits of STN-DBS increase quality of life in chronically levodopa-treated PD patients [4, 5]. While DBS at other targets along motor pathways, such as the internal globus pallidus (GPi), can also be effective for certain manifestations of PD, the STN is the most common target for this intervention [6].

The identification of ideal candidates for STN-DBS is necessary to achieve the best surgical outcomes, given the expense of the procedure and its limited availability in most societies [7, 8]. In general, appropriate patients are selected based on the levodopa challenge test (LCT) which assesses the change in standardized motor ratings that occur in response to the medication [9]. Nonetheless, the accuracy of the LCT as a predictor of the DBS response in individual PD patients has recently been questioned [10]. Alternatives to the LCT and other measures of the dopaminergic response are limited [11, 12].

Network analysis of metabolic brain scans obtained using [^18^F]-fluorodeoxyglucose (FDG) positron emission tomography (PET) may help provide a useful alternative for the evaluation of potential STN-DBS candidates. This general approach has been used to identify and validate disease-specific metabolic networks that are sensitive to the underlying pathological process, which may be modulated by treatment [13, 14]. These topographies, however, are not treatment-specific, and therefore not ideal for assessing targeted therapies such as STN-DBS. Whereas high expression levels for disease networks tend to be associated with worse clinical symptoms, elevated values do not necessarily imply greater treatment efficacy in a given individual.

In this study, we used FDG PET scans from PD patients with STN-DBS on and off stimulation. To identify a specific metabolic brain network induced by treatment, we applied Ordinal Trends Canonical Variates Analysis (OrT/CVA), a form of supervised principal component analysis (PCA), to the scan data obtained for the two stimulation conditions [13–15]. The algorithm isolated a significant STN-DBS-related network (STN StimNet) characterized by consistent increases in expression levels with stimulation in all or nearly all of the subjects and validated in scans obtained in an independent group of STN-DBS patients at a different site. While individual motor outcomes were not used for pattern identification or testing, stimulation-mediated increases in STN StimNet expression correlated with clinical improvement in both groups of STN-DBS patients.

After validation, we examined the relationship of the STN StimNet topography to neural activity in the target region. We observed that STN StimNet expression was primarily driven by theta oscillations at the stimulation target, and the resulting changes in cortical neural activity induced by STN stimulation aligned topographically with the pattern of STN StimNet. In this regard, we found that StimNet expression measured preoperatively using FDG PET or resting-state functional MRI (rs-fMRI) predicted the clinical response to STN stimulation determined after surgery in independent groups of PD patients. In aggregate, the data suggest that preoperative STN StimNet expression plays a significant role in determining an individual patient’s clinical response to stimulation. As such, this functional imaging measure may be useful in selecting optional candidates for DBS surgery.

## Methods

### Patient demographics

We studied 51 PD patients who underwent implantation of DBS electrodes into the subthalamic nucleus (STN). Of these, 41 patients were studied at Site 1 (Northwell Health, Manhasset, NY, USA) and 10 at Site 2 (San Raffaele Hospital, Milan, Italy). Two of Site 1 patients underwent unilateral STN electrode implantation. Demographics and clinical characteristics for the STN-DBS cohorts are summarized in Table S1A. The details of the imaging procedures in each of the cohorts are provided in Supplementary Methods. Ethical permission for the PET studies was obtained from the Institutional Review Board of Northwell Health. Written consent was obtained from each patient after detailed explanation of the procedures.

### Network identification using Ordinal Trends Canonical Variates Analysis (OrT/CVA)

To identify a significant metabolic covariance topography associated with STN-DBS, we analyzed pairs of FDG PET scans acquired on- and off-stimulation (ON and OFF, respectively) on a hemisphere-by-hemisphere basis. This approach leveraged the side-to-side differences in response that characterizes most DBS interventions for PD symptoms [16]. As in previous imaging studies, we sought to identify stable treatment-induced network topographies by maximizing individual responses to the intervention in question [17, 18]. In the current study, we searched for a candidate STN StimNet topography in ON/OFF scan pairs from the 10 hemispheres with the greatest stimulation-mediated motor improvement in the contralateral limbs. The remaining subset comprised the 16 hemispheres with smaller contralateral limb responses, used subsequently for validation. Baseline (OFF) motor ratings were similar for the derivation and validation subsets with contralateral UPDRS limb scores of 10.2±0.8 and 9.6±1.0 (mean±SE), respectively. With STN stimulation, however, the reduction in UPDRS contralateral limb scores differed for the respective subsets, with corresponding changes of 5.6±0.6 (54.6%) and 2.1±0.5 (21.0%) (p<0.001; Welch’s test).

**Figure 1.**
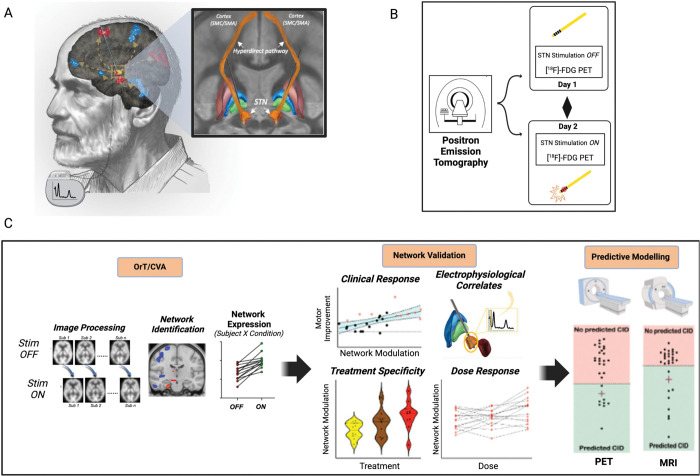
Study design and analytical framework. **(A)** Deep brain stimulation (DBS) electrodes were stereotactically implanted into the subthalamic nucleus (STN) and tunneled subcutaneously via lead extenders to an implantable pulse generator (IPG) placed on the anterior chest wall. *Inset:* 3D representation of typical bilateral deep brain stimulation electrodes (*black*) implanted into the motor portion of the subthalamic nucleus (*orange*) overlaid on adjacent coronal and axial slices from a template image using Lead-DBS [68, 69]. The volume of tissue activated (VTA, *red*), modeled from selected stimulation parameters, active contacts and the surrounding tissue shows involvement of the hyperdirect pathways (*orange tracts*) that connect the motor STN to the ipsilateral sensorimotor cortex (SMC). Adjacent basal ganglia structures anterior to the electrodes (putamen (*maroon*), external globus pallidus (*blue*), and internal globus pallidus (*green*)) are displayed for reference using the DBS Tractography Atlas [70]. **(B)** Schematic representation of data acquisition: Metabolic imaging with [^18^F]-fluorodeoxyglucose (FDG) PET was conducted in PD patients with implanted STN electrodes on two consecutive days after 12 hours of medication washout on each day. On one day, participants were scanned off-stimulation (OFF) after ceasing stimulation for approximately three hours. On the other day, scanning was performed on-stimulation (ON), at the individual’s usual DBS settings. In a subset of patients, scans of regional cerebral blood flow (closely correlated with glucose metabolism) were additionally acquired using [^15^O]-water (H_2_^15^O) PET at multiple DBS settings in a single imaging session (see text). These scans were used to assess the effects of varying stimulation amplitude on network expression*.* **(C)** After image preprocessing, a hemispheric network was identified using a supervised PCA approach applied to the FDG PET images without regard to individual motor outcomes (see Methods). Expression values for the resulting STN stimulation network (STN StimNet) were computed in each subject on a hemisphere-by-hemisphere basis in both the OFF and ON conditions. Further analysis was conducted to examine the relationship of network changes to the clinical response and to electrophysiological recordings from the STN, and to evaluate baseline expression levels computed using FDG PET or resting-state functional MRI (rs-fMRI) as potential predictors of treatment outcome.

Network identification was performed using Ordinal Trends Canonical Variates Analysis (OrT/CVA), a supervised principal component analysis (PCA) (Fig. 1C), discussed in detail elsewhere [13–15]. In contrast to disease-related metabolic networks identified using combined data from patients and healthy subjects, OrT/CVA interrogates scans obtained in individuals under different therapeutic conditions for the presence of significant treatment-induced network topographies. In particular, this approach identified sets of linearly independent (orthogonal) spatial covariance patterns for which individual expression values (principal component (PC) scalars) change consistently with treatment in all or nearly all subjects [15, 16, 18–21]. Notably, the identification of significant network topographies by this approach does not require individual subject clinical response data, e.g., ON or OFF UPDRS ratings or treatment-mediated changes in these ratings. In this way, OrT/CVA avoids leakage of outcome information into the training set and consequent overfitting errors. Rather, the algorithm interrogates the data for the presence of monotonic changes (increases or decreases) in pattern expression with treatment [19, 21]. The details of network identification and validation have been described in detail elsewhere [15, 16, 22] and expanded in Supplementary Methods.

### Network validation

#### Clinical correlations:

Expression values (subject scores) for the STN-DBS-related network (STN StimNet) were computed in prospective scan data using a previously described software routine available at www.feinsteinneuroscience.org [23]. In this study, STN StimNet scores were computed separately for each hemisphere/implanted electrode, as well as for the whole brain. For comparison of STN StimNet expression values across groups, values were *z*-scored to an age- and sex-matched healthy control (HC) sample (n=33).

For validation, we computed STN StimNet expression values in the Site 1A hemispheres (n=16) not used for network identification. For testing, we evaluated corresponding hemisphere values from the independent Site 2 sample (n=10). In both groups, we evaluated the relationship of the stimulation-mediated network changes to individual clinical outcomes as determined by corresponding changes in UPDRS motor ratings. For Site 1, network correlations were assessed for changes in contralateral limb ratings (sum of limb subscales for tremor, akinesia, and rigidity) and for composite whole-body motor scores. UPDRS limb ratings were not available for the Site 2 patients. Therefore, the network correlations in this group were assessed with whole-body scores. For both groups, clinical–network correlations were evaluated by computing Pearson product-moment correlation coefficients, which were considered significant for p<0.05.

### Prediction of DBS outcomes using baseline STN StimNet expression levels

To evaluate STN StimNet expression as a potential predictor of the clinical response to STN-DBS in PD patients, we examined the correlation between treatment-mediated improvement in motor ratings and baseline network measurements obtained OFF stimulation in patients with implanted STN electrodes. Relationships between the clinical response and OFF-state network expression were separately assessed in STN-DBS patients from Site 1 (Site 1A, n=13). (One Site 1 A patient was excluded from this part of the analysis because of disproportionate influence on the regression model as determined by Cook’s distance [24].) Similar relationships were assessed from Site 2 (n=10). A separate group of Site 1A patients (n=12) underwent FDG PET an average of 8.7 months prior to their DBS surgery.

Lastly, we explored the possibility of measuring STN StimNet expression levels in rs-fMRI fALFF images acquired an average of 12 days prior to STN-DBS surgery in a subgroup of Site 1 patients (Site 1B, n=15). STN StimNet expression levels measured in OFF in patients from Site 1A and Site 2, and preoperatively for patients from Site 1A and B, were correlated with stimulation-mediated motor changes recorded after surgery (see text), and were considered significant for p<0.05, Pearson correlations. To assess the interactions of other clinical variables with preoperative network expression in relation to motor outcome, we performed multiple linear regression analysis of the corresponding data from the Site 1B rs-fMRI sample, with relative stimulation-mediated improvement in UPDRS motor ratings $\left(\frac{OFF-ON}{OFF}\right)$ as the dependent variable in the model. In addition to preoperative STN StimNet expression, we used the preoperative LCT, which measures the relative response of UPDRS motor ratings to oral levodopa/carbidopa, as a predictor, as well as disease duration. To partition the predictive power of each predictor based on its marginal contribution to the dependent variable, we additionally calculated Shapley values for each of the predictor variables [25].

### Electrophysiological recordings

To understand the electrophysiological basis for preoperative STN StimNet expression as a predictor of the clinical stimulation response, we collected local field potential (LFP) data from the STN in 14 of the 15 members of the Site 1B. These individuals were bilaterally implanted with the Percept^™^ PC DBS system (Medtronic, Minneapolis, MN, USA), which allows for the recording of LFPs from the STN target under physiological conditions comparable to the preoperative StimNet measurements. Recordings from the devices were performed postoperatively in the medication off state, prior to initiation of stimulation. (One member of this group had a DBS device that did not permit chronic recording and was therefore excluded from this portion of the analysis). Analysis of electrophysiological data was performed in MATLAB R2023a (MathWorks Inc., Natick, MA, USA). For power spectral analysis, the data was analyzed across electrode contacts in the STN that produced the greatest motor benefit. Data streams were sampled at 250 Hz, and a fast Fourier transform (FFT) was applied in 1 Hz steps. Frequency bands of interest were defined as delta (1–4 Hz), theta (5–8 Hz), alpha (9–12 Hz), low beta (13–20 Hz), high beta (21–30 Hz), and gamma (31–100 Hz). For each electrode, power across each frequency band was computed as the integral across the associated frequency range, which was normalized to the power across the entire frequency range (1–100 Hz).

Two additional patients underwent intraoperative recordings unilaterally using the Alpha Omega Neuromodulation Targeting System (Alpha Omega Engineering, Israel). Simultaneous LFPs were recorded along with electrocorticography (ECOG) using a 1 × 8 channel subdural grid (4.5 mm contact diameter, 10 mm intercontact spacing) placed over the cortical surface that was accessible from the cranial burr hole entry used for DBS implantation. ECOG data were also acquired during active subthalamic stimulation at 0 and 2 mA, 160 Hz and 60 μs. For cortical gamma, the analysis was restricted to the finely tuned gamma (FTG) range (60–90 Hz). Recordings were resampled to 1 kHz, and bandpass filters (order 5, width 4 Hz) were applied to 60 Hz (and resonant frequencies) to mitigate line noise. A morlet wavelet (filter width 8; 1 Hz frequency steps) was applied to the entire stimulation and baseline epochs. The resulting power spectrum was *z*-score corrected to the baseline period (for each channel and stimulation trial) to allow for comparisons across contacts and participants. For each contact, gamma power was computed as the mean across the stimulation epoch and gamma frequency range. Phase-amplitude coupling (PAC) analysis in the baseline (off stimulation) condition was quantified using the modulation index (MI) for theta-band (5–8 Hz) phase activity in the subthalamic nucleus (STN) and the FTG (60–90 Hz) amplitude activity recorded from each subdural grid contact. MI values were calculated according to the method of Tort et al. (2010), which provides a measure of the extent to which the phase of the STN theta oscillations modulated cortical gamma activity under baseline conditions [26]. Changes in stimulation mediated FTG power and baseline MI were correlated with corresponding regional loadings (i.e., the average voxel weights associated with each subdural contact) on the STN StimNet topography. (Due to limited sampling, MI for one subdural contact was excluded as an outlier due to the disproportionate influence on the regression equation.) STN and Cortical electrode were localized intraoperatively using the O-Arm Surgical 3D imaging system (Medtronic, Minneapolis, MN, USA). Images were sequentially registered to preoperative MRI and spatially normalized to MNI space using the ANTs function within 3D Slicer. The weight on STN StimNet for a given contact, which measures the local contribution to the network from the adjacent brain, was represented by the average voxel weight (*z*-scored) in an 8 mm sphere placed in the cortical area located directly beneath corresponding contact.

### Predictive model of clinical outcomes based on preoperative network expression

Lastly, we demonstrated how preoperative STN StimNet expression can be used to identify those PD patients who are likely to benefit most from STN-DBS surgery. We stratified potential STN-DBS-related outcomes according to the clinically important difference (CID), a method that links quality of life and other patient-reported outcomes to specific thresholds on the clinical rating scale [27]. By relating treatment-mediated changes in ratings to the patients’ perceived outcome, CID provides an index of clinical significance that is not available otherwise. With invasive surgical procedures such as STN-DBS, which carry patient risk and societal expense, CID should ideally be larger than for alternative pharmacological or other non-invasive interventions [28]. Prior analysis of blinded PD studies has shown that large CID equates to an absolute change of 10.8 in total UPDRS motor ratings [27]. To estimate the STN StimNet expression score that corresponds to a large predicted CID response with STN stimulation, we used the regression model presented in the text (Fig. 5B). To this end, we analyzed FDG PET scans from 175 Site 1 PD patients, who were divided into roughly 4-year blocks based on the time from clinical diagnosis [29–31]. For each patient, we computed whole-brain STN StimNet expression and standardized the measurements with respect to corresponding values from the reference group of 33 healthy control subjects (see above) [32]. We compared the resulting values to groups of patients considered not to be DBS candidates for clinical reasons described in detail in Supplementary Methods and Table S1B. Network expression values were compared across the various groups using one-way ANCOVA, with age as a covariate and appropriate post-hoc contrasts.

## Results

### Network identification

**Figure 2.**
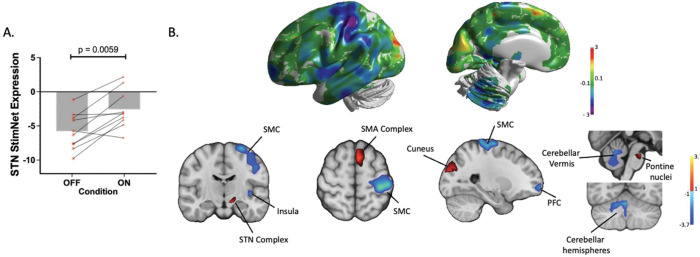
Identification of the STN StimNet metabolic topography. **(A)** STN StimNet expression values showed increases in nine of the 10 hemispheres used to identify the network (p=0.0059; permutation test) (see text). **(B)** 3D brain surface views of the metabolic topography of the STN stimulation-related network (STN StimNet). Coronal, axial, and sagittal views of a T1W MRI template showing areas with significant hemispheric contributions to the STN StimNet topography (Table 1). The network was characterized by stimulation-mediated increases (*red*) in the subthalamic complex (STN/substantia nigra), supplementary motor area (SMA), pons and cuneus, and reductions (*blue*) in the sensorimotor cortex (SMC) and cerebellum (vermis and paravermian structures). [Regions were displayed on the corresponding slices if the corresponding voxel weights contributed significantly to the network (|z| ≥ 1.0) and were considered reliable on bootstrap estimation (inverse coefficient of variation (ICV) |z| ≥ 1.65, p<0.05; 1000 iterations).]

The details of DBS electrode placement and the study design are illustrated in Figure 1 (see Methods). To identify a simulation-specific spatial covariance pattern in the Site 1A data, we applied OrT/CVA (see Methods) to FDG PET scans acquired on and off STN stimulation in the 10 hemispheres with large therapeutic responses in the contralateral limbs. A significant ordinal trend, i.e., an increase in hemispheric network expression with stimulation in all or nearly all subjects (see Methods) was detected (Fig. 2A), with stimulation-induced increases in pattern expression in 9 of 10 hemispheres (p=0.0059; permutation test). The topography of the resulting STN-DBS-related metabolic network, termed STN StimNet (Fig. 2B), was characterized by stimulation-induced metabolic increases (*red clusters*) in the subthalamic complex (STN/substantia nigra), supplementary motor area complex (SMA, BA6), cuneus (BA19), and dorsal pons. These changes were accompanied by stimulation-induced reductions (*blue clusters*) in the sensorimotor cortex (SMC, BA4/3), prefrontal cortex (BA9) and insula, and in the cerebellar vermis and hemispheres (lobules II, IV, V and the medial aspect of lobule VI) (Table 1). Voxel weights in the respective clusters were found to be reliable by bootstrap resampling (|*z*| ≥ ±1.64, p<0.05, one-tailed; inverse coefficient of variation (1000 iterations)).

**Figure 3.**
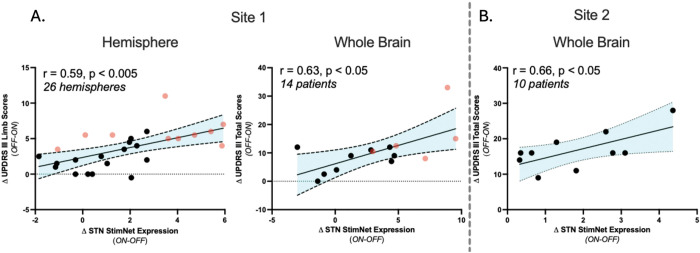
Stimulation-related changes in STN StimNet expression correlate with concurrent improvement in motor ratings. **(A)** *Left*: In the data from Site 1A (see Methods), stimulation-mediated changes in hemispheric network expression ipsilateral to the electrode correlated with improvements in contralateral limb UPDRS motor ratings (r=0.59, p<0.005; Pearson correlation). *Right*: An analogous correlation was seen in the same subjects for whole brain network expression with whole-body motor ratings (r=0.63, p<0.05). [Values from scans used for network identification (see text) are displayed by red symbols; those used for in-sample testing are displayed by black symbols.] **(B)** For independent testing, we computed whole brain STN StimNet expression values ON and OFF stimulation in the independent Site 2 dataset (see text). As in Site 1, changes in these values correlated with improvement in total motor UPDRS ratings (r=0.66, p<0.05).

Next, we next prospectively computed STN StimNet expression on- and off-stimulation in the 16 remaining hemispheres in the Site 1A cohort (i.e., those not used for network identification), and a significant ordinal trend was confirmed for the whole sample, i.e., Site 1A cohort (p=0.0005; permutation test). Individual treatment-mediated changes in motor ratings were not employed by the algorithm for network identification or testing (see Methods). We, nonetheless, observed significant correlations between stimulation-mediated increases in STN StimNet expression and concurrent improvement in contralateral limb motor ratings (r=0.59, p<0.005; Pearson correlation, two-tailed) (Fig. 3A, *left*), and between corresponding changes in whole-brain network expression and whole-body motor ratings (r=0.63, p<0.05) (Fig. 3A, *right*). The stability of these relationships was supported by the excellent test-retest reliability (ICC=0.97) of the STN StimNet subject scores over an 8-week interval in PD patients (n=22) (Fig. S1).

Lastly, we determined whether the observed changes in STN StimNet expression were reproducible in an independent group of PD patients (Site 2) who had likewise undergone STN-DBS surgery and were scanned with FDG PET in the on- and off-stimulation conditions on consecutive days (See Methods). In this patient sample, significant stimulation-mediated increases in network expression were observed (p<0.05; Student’s paired *t*-test, two-tailed), which correlated with concurrent improvements in total motor ratings (r=0.66, p<0.05; Pearson correlation, two-tailed) (Fig. 3B).

### Electrophysiological correlates of network activity

To study the network dynamics of STN StimNet, we recruited an additional group of PD patients (rs-fMRI cohort; see Methods) with implanted DBS devices that permitted recordings of STN local field potential (LFP) outside of the operating room in naturalistic settings (Fig. 4A). LFP recordings from STN (28 hemispheres) were obtained in the off condition. Analysis of these data revealed a significant correlation between theta-band power (5–8 Hz) in the STN and measurements of STN StimNet expression obtained preoperatively in the Site 1B cohort using rs-fMRI (r=0.4, p<0.05; Pearson correlation) (Fig. 4B). Indeed, STN theta-band power also correlated positively (*yellow lines*) or negatively (*green lines*) with rs-fMRI fALFF measurements of neural activity at key STN StimNet nodes (Fig. 4C).

It is known that exaggerated power in the beta frequency band (13–30 Hz), especially within the low-beta range (13–20 Hz), is a marker of motor disability in PD [33, 34]. Even so, a significant relationship was not observed between STN low-beta power and STN StimNet expression (r=−0.25, p=0.2; Pearson correlation). Notably, power in this pathological frequency band was found to correlate with contralateral limb motor ratings (r=0.39, p<0.05; Pearson correlation) (Fig. 4D).

Focal stimulation in the STN modulates distant interconnected cortical regions by entraining neuronal activity in the finely tuned gamma range (FTG, 60–90 Hz) [35]. To gain insight into the effects of STN stimulation on neural activity recorded in cortical network regions, we measured stimulation-mediated changes in cortical FTG in two patients who underwent intraoperative STN macrostimulation (2 mA) with simultaneous electrocorticography (Fig. 4E). Stimulation mediated changes in FTG power (16 contacts; see Methods) correlated with the magnitude and sign of the corresponding regional loadings (voxel weights) on the STN StimNet pattern (r=0.5, p<0.05) (Fig. 4F). Likewise, an analogous correlation with cortical loadings on the network was observed for the baseline (OFF stimulation) Modulation Index (MI; see Methods), a measure of the influence of baseline STN theta-band power on stimulation-mediated FTG responses in corresponding brain regions (r=0.53, p<0.05) (Fig. 4G). These findings point to a direct relationship between stimulation responses in downstream STN StimNet regions and their respective contributions to overall network activity.

### Baseline network expression predicts stimulation-mediated motor benefit

Given the significant relationship of the STN StimNet topography to stimulation-mediated modulation of neural signals in cortical regions, we explored the possibility that motor outcomes are determined to a degree by individual patient differences in baseline expression levels for this network. Firstly, we determined whether an association was present between STN StimNet expression measured in FDG PET scans acquired off-stimulation (OFF) and improvement in motor ratings in the original DBS sample used for network characterization (Site 1A) and subsequently in the independent validation sample (Site 2). Indeed, significant correlations between these measures were found in the data from both sites (Site 1: Hemisphere: r=−0.52, p<0.01 (Fig. S3A); Whole brain: r=−0.66, p<0.02 (Fig. S2A); Site 2: Whole brain: r=−0.68, p<0.05 (Fig. S2B, *right*)).

**Figure 5.**
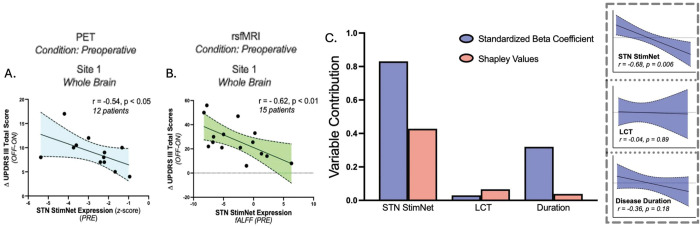
Preoperative STN StimNet expression predicts motor outcomes following DBS surgery. **(A)** To assess predictions of stimulation-mediated motor improvement based on preoperative (PRE) STN StimNet expression, we measured network values in 12 other patients (Site 1A) who underwent FDG PET imaging an average of 8.7 months prior to bilateral STN-DBS surgery (see text). A significant correlation was seen between these measures (r=−0.54, p<0.05). **(B)** To determine whether similar predictions were feasible using non-invasive rs-fMRI methods, we measured STN StimNet expression fALFF maps obtained in an independent group of 15 patients (Site 1B) who were scanned an average of 12 days before bilateral STN-DBS surgery (see text). As with the PET cohort, preoperative STN StimNet expression correlated with stimulation-mediated motor improvements measured an average of 5 months after surgery (r=−0.62, p<0.05). **(C)** *Left:* Comparison of preoperative STN StimNet expression and the levodopa challenge test (LCT) as predictors of the motor response to STN stimulation in a multiple regression model that includes disease duration (see text). The analysis revealed preoperative STN StimNet expression to be the most accurate of the baseline predictors, as evident from the relative size of its regression coefficient (β=−0.83, p<0.02; *blue bar*). Examination of Shapley values (see text) for the predictor variables showed a similar trend, with high values associated with preoperative network expression (0.42) compared to LCT and disease duration (0.10 and 0.05, respectively). This indicated that STN StimNet shares predictive information with the other variables. *Right:* Partial correlation leverage plots of each of the three predictor variables were constructed to isolate the effects of each measure while controlling for the others. Partial correlations were significant only for preoperative STN StimNet expression but not for the other predictors.

Given the findings in OFF, we then determined whether accurate predictions of the motor responses were observed using FDG PET scans obtained preoperatively. Thus, in 12 other patients (Site 1) preoperative network expression values (average of 8.7 months before DBS surgery) correlated with motor benefit recorded 17.3 months after surgery (Hemisphere: r=−0.73, p<0.0001 (Fig. S2B), Whole brain: r=−0.54, p<0.05 (Fig. 5A)). Lastly, we determined whether analogous predictions of stimulation-mediated motor outcome were possible using preoperative STN StimNet expression values obtained using rs-fMRI. To this end, we analyzed rs-fMRI data from 15 PD patients scanned an average of average 12.4 days prior to STN-DBS surgery. STN StimNet expression in these patients were computed using the fractional amplitude of low frequency fluctuations (fALFF) derived from the original time-series data (see Methods). As with FDG PET, preoperative measurements of network expression correlated with independent measures of motor benefit obtained an average of 6.3 months after surgery (Whole brain: r=−0.66, p<0.01 (Fig. 5B)).

Although the LCT is regarded as a predictor of the DBS response, other variables such as disease duration can influence the accuracy of the measure [36]. We found that entering duration into the model, along with preoperative STN StimNet expression and relative improvement in motor ratings on the LCT, accounted for a sizeable amount of between-subject variation in the DBS motor outcomes (F(3, 11)=4.182, p=0.033, R^2^=0.53; multiple linear regression (Fig 5C)). Interestingly, the prediction of stimulation-mediated motor improvement was driven mainly by subject differences in preoperative STN StimNet expression (β=−0.83, p<0.02), either directly or by sharing predictive information with other variables (Shapley value = 0.4) (Fig. 5C).

Next, given the importance of accurate lead placement in DBS outcome, we determined whether predictions of stimulation-mediated motor benefit are enhanced by the addition of structural information regarding the proximity of the electrode to the STN “sweetspot” and corresponding estimates (see Methods) of the volume of tissue activated (VTA) (Fig. S5B) [37]. Indeed, the combination of preoperative StimNet expression and postoperative lead location accurately predicted the relative motor improvement with STN-DBS (F(2, 12)=6.742, p=0.011, R^2^=0.53; multiple linear regression). Moreover, the contribution of network expression to the model remained significant (β=−0.52, p<0.05) after controlling for the effects of electrode placement (β=0.31, p=0.2).

### Preoperative network expression can be used to select optimal surgical candidates for STN-DBS

**Figure 6.**
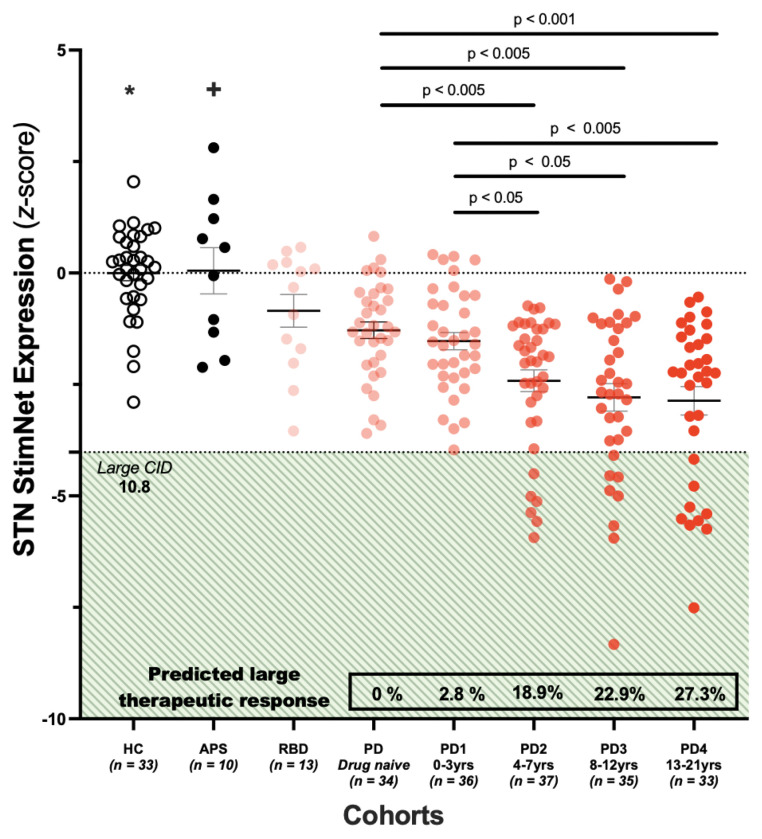
Using STN StimNet expression to select optimal PD patients for DBS surgery. To illustrate the potential use of baseline STN StimNet expression as a predictor of the clinical response to stimulation, we computed this measure in FDG PET scans from 175 PD patients with disease duration, i.e., time from diagnosis, ranging from 0 to 21 years (see text). We found that network expression declined with increasing duration (p<0.001; ANCOVA, corrected for age). Low baseline expression levels, denoting greater likelihood of substantial clinical benefit from STN stimulation, became prominent after the first two 4-year duration blocks, without further decline in subsequent blocks (p>0.9; post-hoc comparisons, Bonferroni correction). Based on the regression model in Figure 5B, the percentage of patients predicted to have a meaningful response to STN stimulation, as judged by a large clinically important difference (CID), i.e., a treatment-mediated reduction in UPDRS motor ratings of at least 10.8 points (see Methods), began to increase approximately 4 years from the time of clinical diagnosis. Accordingly, the percentage of optimal DBS candidates rose from 18.9% in the second block (4–7 years) to 27.3% in the final block (13–21 years). Of note, optimal DBS candidates of this sort were not seen in patients with autopsy-confirmed atypical parkinsonian syndrome (APS), individuals with REM sleep behavior disorder (RBD), or in drug naïve early-stage PD patients (see text). These conditions are thought to be either refractory or too early for DBS surgery. *Baseline STN StimNet scores was reduced in the PD population compared to matched healthy control subjects (F(5,195)=17.937, p<0.001; one-way ANOVA, adjusted for age). +While network expression values in autopsy-confirmed APS patients were not significantly different than the healthy control (HC) group, expression values in this group were elevated relative to PD patients scanned after the four-year mark (adjusted p<0.001; post-hoc Bonferroni tests). This suggests that few if any APS patients will have meaningful clinical benefit in response to DBS surgery.

Given that STN-DBS has been proposed as a treatment option in the earlier stages of PD [38], we considered using preoperative STN StimNet expression to select the patients who are most likely to achieve optimal benefit from the procedure. To show how these measurements might be used, we prospectively computed STN StimNet expression in 175 PD patients who were scanned with FDG PET between 0–21 years after the time of diagnosis (Fig. 6). The patients were grouped into blocks according to disease duration. Those with short and intermediate duration were divided into roughly three 4-year blocks (0–3 years, 4–7 years, and 8–12 years); those with long duration were grouped into a single 8-year block (13–21 years) (see Methods). By entering STN StimNet expression in individual patient scans into the regression equation that relates the preoperative network measure to stimulation-mediated motor benefit (Fig. 5B), we estimated a hypothetical treatment response for each subject and duration block. The predicted motor outcomes were then used to estimate the percentage of individuals in each block in whom the clinical response was expected to be large enough to justify the procedure (see Methods). The results in PD patients were compared to groups of individuals not typically considered to be DBS candidates, such as patients with atypical parkinsonian syndromes (APS) (n=10), who are generally refractory to DBS surgery, treatment-naïve early stage PD patients (n=34) whose symptoms are typically too mild to warrant surgery as an initial option, and individuals with REM sleep behavior disorder (n=13), a preclinical syndrome in which parkinsonian manifestations have yet to appear. We found that STN StimNet expression was reduced in the chronically treated PD groups (PD1-PD4) compared to healthy control subjects (F(5,195)=17.937, p<0.001; one-way ANCOVA, adjusted for age). Analysis across duration blocks revealed lower expression levels with advancing PD (F(3,136)=4.983, p<0.005; one-way ANCOVA, adjusted for age p<0.001). Notably, baseline expression values did not decline significantly after the second PD block (duration 4–7 years) (adjusted p>0.9; post-hoc Bonferroni tests). By the same token, the proportion of patients expected to have substantial clinical improvement in response to STN-DBS (defined as a large (>10.8 point) clinically important difference (CID); see Methods) rose incrementally from 2.4% for 0–3 years to 27.7% for 13–21 years (p<0.05, Fisher’s exact test). By contrast, baseline STN StimNet expression did not differ from normal in autopsy-confirmed APS patients (p>0.9; post-hoc Bonferroni tests) or those with RBD (p>0.5, post-hoc Bonferroni tests), consistent with low likelihood of substantial benefit in these groups. No patients in these preclinical/early-stage PD groups had STN StimNet expression of sufficient magnitude for a large CID stimulation response, and as such DBS surgery would not be justified. By contrast, patients likely to exhibit a suitably large response are encountered with increasing frequency beginning at the four-year mark.

## Discussion

In this study, we identified and validated STN StimNet, a network biomarker of the response to STN-DBS in PD patients. This network was treatment-related in that it demonstrated greater modulation with STN stimulation than other antiparkinsonian interventions (Supplementary Results, Fig. S1). As a treatment marker, changes in STN StimNet expression correlated with motor benefit in two independent groups of PD patients with implanted STN electrodes. Moreover, STN StimNet expression was driven by the intensity of stimulation at the target (Supplementary Results, Fig. S4), while fALFF measurements of neural activity obtained preoperatively in downstream network regions correlated with off-stimulation STN theta-band power recorded in the same subjects after DBS surgery. The relationship between preoperative StimNet expression and off-stimulation LFP recordings from the STN suggests that DBS outcomes are determined at least in part by baseline differences in network activity. Indeed, a consistent predictive relationship was observed between preoperative expression levels measured with FDG PET or rs-fMRI and stimulation-mediated motor outcomes recorded several months after DBS surgery.

Topographically, STN StimNet had significant contributions from cortical and subcortical motor regions found previously in relation to therapeutic stimulation at this site [39–41]. We note that STN StimNet exhibited only marginal spatial homology with the previously validated Parkinson’s disease-motor-related pattern (PDRP [13, 42]) (Fig. S6). In keeping with earlier studies [43], stimulation-mediated improvement in motor ratings in the current data correlated with reductions toward normal in PDRP expression (Fig. S7A). While a modest correlation was present between the latter changes and those for STN StimNet (Fig. S7B), the PDRP network response is not specific for any one intervention [14, 15, 44, 45]. Likewise, different relationships between subject expression and disease duration were noted for the two networks (Fig. S7C, *inset*). In contrast to the stepwise increases in PDRP expression seen with increasing disease duration, STN StimNet values began to approach a floor four years after diagnosis [29, 30].

The observed differences in expression values for the two networks can be explained by their spatially distinct topographies. STN StimNet comprised a group of connected brain regions that were differentially modulated by stimulation. This topography differed from PDRP, which included regions directly associated with the underlying disease process [45–47]. These findings were further supported by measurements of STN low-beta activity, a known driver of PD motor impairment [48]. While abnormal, this frequency band did not correlate with baseline STN StimNet expression levels.

Of note, the STN StimNet topography include regions with negative voxel weights (*blue clusters* in Fig. 2B) localized to the sensorimotor cortex (SMC) and the contralateral cerebellar hemisphere and vermis, consistent with stimulation-induced reductions as part of the network. Elements of these regions also contribute to the PDRP (*yellow clusters* in Fig. S6), representing areas of relative increase in metabolic activity that occur as part of the disease [49, 50]. These areas are important as likely mediators of PD motor symptoms, including tremor [16, 51]. By the same token, the STN StimNet also contains regional elements with positive weights (*red clusters* in Fig. 2B), corresponding to areas in which STN stimulation increased local metabolic activity independently of the underlying disease process. While high frequency stimulation is hypothesized to inhibit the overactive STN, alternative observations have suggested that an increase in output from the target region may constitute to an “informational lesion” that is created by DBS [52, 53]. In this regard, the stimulation-mediated increases that we observed in the subthalamic complex (the STN and adjacent substantia nigra) and in its projection ventrally to the pontine nuclei are consistent with the changes in local cerebral blood flow and metabolism with DBS reported in earlier PET studies [54, 55]. Importantly, positive region weights on the network were also observed in the SMA complex (preSMA and SMA) [56], driven perhaps by antidromic stimulation of the hyperdirect pathway [57–59]. The contribution of the SMA complex to the STN StimNet topography is compatible with recent observations showing that improved functional connectivity in pathways linking the aforementioned regions with the STN stimulation target is associated with greater relief of PD motor symptoms, especially akinesia and rigidity [41, 59, 60].

The recordings of LFPs in STN acquired through closed loop DBS provided unique information regarding the neural basis of STN StimNet activity. Recent evidence has suggested that low frequency activity, particularly in the theta range, is critical for inter-regional neural communication within spatially distributed cortical networks [61, 62]. In keeping with this hypothesis, we found that STN StimNet activity measured in preoperative rs-fMRI fALFF images correlated with STN theta power (Fig. 4B). Likewise, local contributions from key nodes to overall network activity (represented by the magnitude of the corresponding voxel weights) were also driven by STN theta. This effect was most pronounced in the GPi, which receives direct glutaminergic input from STN [57]. Indeed, of the major nodes of STN StimNet, GPi exhibited the strongest correlation between local fALFF activity and STN theta power.

Relationships with the STN theta power may also explain the direction of the local changes, i.e., increases or decreases in regional activity with focal stimulation, based on the sign of the corresponding voxel weights. Thus, the cortical nodes modulated most by STN theta activity (high MI) were predicted to exhibit the greatest metabolic increases after stimulation (red clusters on STN StimNet). Such effects may be attributable to spread of current along the white matter pathways that connect these regions to the STN through one or two synapses [63]. By contrast, the regions modulated least by STN theta activity (low MI) may have strong functional connectivity with the STN, but act through multiple synaptic relays which can give rise to differential stimulation effects [64]. Such regions may in fact exhibit metabolic reductions with stimulation (*blue* clusters on STN StimNet), reflecting mainly effects occurring downstream from the stimulation target.

An unexpected finding in this study was the correlation between STN StimNet expression measured in the OFF condition and the stimulation-mediated motor response (Site 1A and Site 2). In both groups, the patients with the lowest network expression values off stimulation experienced the largest increases in expression during STN stimulation, and accordingly the greatest clinical benefit. Correlations with motor outcome were also seen for network expression measurements obtained *preoperatively* with FDG PET or rs-fMRI (Site 1A and B). As with STN StimNet expression in the OFF condition, the lowest preoperative measurements, whether obtained with FDG PET or rs-fMRI, were associated with the best outcomes. It is also noteworthy that analogous predictions of clinical outcome were not observed with PDRP expression values measured after surgery in the OFF condition or preoperatively.

While disease modification effects have been attributed to STN-DBS, the intervention is largely symptomatic: efficacy is gauged by improvements in standardized motor ratings, which may or may not be clinically meaningful. This is particularly relevant to surgical interventions such as STN-DBS, in which the inherent risks of invasive electrode implantation and the attendant cost of the procedure need to be leveraged against the clinical benefit provided to the individual patient [27]. Conversely, given the limited availability of DBS procedures and related services, even in affluent societies, the identification of optimal surgical candidates, i.e., those patients likely to derive the greatest benefit from the intervention, becomes an important consideration [11]. The current study suggests that preoperative measurements of STN StimNet expression may be helpful in identifying such patients while excluding individuals less likely to benefit from DBS surgery (Fig. 6). In routine clinical practice, the LCT (≥ 33 % improvement in motor symptoms with oral levodopa/carbidopa) is considered the gold standard for appropriate patient selection [65]. While the LCT is undoubtedly valuable in confirming the diagnosis of idiopathic PD and assessing candidacy for DBS, its accuracy as a predictor of individual motor improvement has been questioned [10, 66]. To maximize the number of years of improved quality of life enjoyed by PD patients undergoing DBS procedures, treatment paradigms have recently shifted towards earlier surgery [38]. That said, predictions of DBS outcome based on the LCT have been found to be less in PD patients with shorter disease durations and more pronounced long-duration clinical responses to levodopa [36]. Our comparison of preoperative STN StimNet expression and the LCT as predictors of the STN-DBS treatment response was limited to the 15 patients in the Site 1 rs-fMRI sample. That said, the analysis revealed baseline network expression to be the stronger predictor of motor outcome. This is attributable in part to shared contributions of the two measures in the multiple regression model by the moderate size of the Shapley value. A predictive study is needed in a larger patient sample to determine the accuracy of predictions made based on the two preoperative markers, singly or in combination.

That said, appropriate lead placement is fundamental to the outcome, given that a misplaced or sub-optimally placed electrode is unlikely to be effective no matter of how well-suited the patient is for the procedure. In this regard, STN StimNet may have utility in the management of treatment failure after DBS surgery. If a good outcome is predicted based on preoperative network expression, one may wish to re-adjust the DBS tuning parameters, or even reposition the leads – given that errors of as little as 2 mm can have substantial effects on clinical outcome [67].

The current study has some limitations. The scans were performed for research as opposed to clinical purposes, which limited the size of the available datasets. Along these lines, to identify a network relating to individual electrodes, the analysis was done on a hemisphere-by-hemisphere basis. While the behavior of each electrode was lateralized with regard to symptom relief, ON-state imaging was conducted at both sites with simultaneous deployment of the electrodes in both hemispheres. This strategy was pursued because of radiation safety considerations which prohibited acquiring ON scans in one hemisphere independently of the other. We recognize that our approach can potentially introduce confounding effects of stimulation-mediated spill-over from the opposite side. Nonetheless, we found that STN stimulation effects were sufficiently lateralized such that each side functioned independently with respect to hemispheric network modulation and changes in contralateral limb motor ratings. Indeed, relationships between hemispheric STN StimNet expression and contralateral limb motor outcome were maintained when corresponding measurements were computed for the whole brain.

The proportion of patients with large CID responses to DBS was estimated from baseline STN StimNet expression scores using a linear regression equation fitted to the Site 1 data (Fig. 5B). This sample was small, and the substantial variability that was present may influence predictions made based on individual patient preoperative network expression. Larger patient samples will be needed for more accurate and reproducible predictions of DBS outcome based on these preoperative network expression measurements. We additionally recognize that inferences from the electrocorticography data must be taken with caution, given that the recordings were from a small portion of the cortical surface related to STN StimNet. Future studies with broader sampling over the cortex will be needed to confirm these findings.

In conclusion, we describe a novel functional brain network that is modulated by STN stimulation. By recording STN LFPs after DBS surgery, we found that network activity correlated with theta-band oscillations at the target site, consistent with enhanced synchronization of neural activity in connected regions. In this regard, we found that preoperative measurements of network expression obtained with FDG PET or rs-fMRI correlated consistently with the clinical response to STN stimulation in PD patients. The results suggest the potential utility of this network in the surgical candidates most likely to obtain optimal benefit from the procedure.

## Supplementary Material

This is a list of supplementary files associated with this preprint. Click to download.

UnadkatBrainSupplMat.docx

## Figures and Tables

**Figure 4.** Relationship of STN StimNet to local field potentials recorded at the surgical target. **(A)** 14 PD patients with bilaterally implanted electrodes had implantable pulse generators (IPGs) that allowed for recording of local field potentials (LFPs) from the motor STN. To facilitate comparisons with rs-fMRI measurements of STN StimNet expression, these recordings were performed outside the operating room, under naturalistic conditions. **(B)** Activity in the low frequency bands, particularly the theta range, facilitates inter-regional communication and network synchronization (see text). In the Site 1B sample, we found that localized STN theta band activity (5–8 Hz) recorded in the off stimulation (OFF) condition correlated with StimNet expression values computed in fractional amplitude of lowfrequency fluctuations (fALFF) data (r=0.40, p<0.05). **(C)** Further, the theta band of STN had differential effects on neural activity at major STN StimNet regions. The internal segment of the globus pallidus (GPi), located downstream from STN is a major target of excitatory output from the latter structure. Neural activity in GPi exhibited a moderately strong correlation with STN theta activity (r=0.61, p<0.001, Pearson correlation). **(D)** By contrast, power in the STN beta band, particularly in the low beta (13–20 Hz) range is thought to be a major driver of the clinical manifestations of PD [48]. Indeed, activity in this frequency band correlated with contralateral limb motor ratings (r=0.39, p<0.05). Nonetheless, no significant correlation was observed between STN low beta power and STN StimNet expression levels (p=0.2). **(E)** To determine the relationship between the physiological changes in cortical regions during STN stimulation and LFPs from the STN, we simultaneously obtained intraoperative ECOG recordings from the cortex (see Methods). Electrode contacts (*spheres*) were mapped in MNI space and are color-coded by their respective STN StimNet weights: cortical loci with relatively large contributions to overall STN StimNet activity (ïregion weights÷^3^ 1.0) are represented by *dark red* (region weights ^3^ +1.0) or *dark blue* (region weights £ - 1.0); loci with smaller contributions to the network (ïregion weights÷ £ 1.0) are represented by lighter shades of these colors. **(F)** Active stimulation during DBS has been shown to modulate cortical regions through neural entrainment in the gamma range (60–90 Hz) [35]. Stimulation-mediated changes in this finely tuned gamma range correlated with region weights on STN StimNet in corresponding cortical areas (r=0.50, p<0.05). **(G)** The influence of STN theta activity on cortical dynamics was measured by the Modulation Index (MI) at each of the contacts (see Methods). A significant correlation was noted between MI and the magnitude and sign of the STN StimNet region weights for the corresponding cortical areas (r=0.53, p<0.05).

**Table 1.** Regional topography of STN StimNet

Region	MNI Coordinates *Peak Voxel*			Region Weights *Peak Voxel* (z-score)
	x*	y	z	
*STN stimulation mediated metabolic increases (ON > OFF)*				
Subthalamic complex (STN)^a^	±12	−16	−14	2.63
Dorsal pons	±2	−20	−24	3.13
Supplementary motor area complex (SMA) (BA6)^b^	±2	16	54	3.71
Cuneus(BAe19)	±20	−98	20	3.11
*STN stimulation mediated metabolic decreases (ON < OFF)*				
Cerebellar vermis and hemispheres (lobules II, IV, V and the medial aspect of lobule VI)	±2	−62	−20	−3.55
Sensorimotor cortex (SMC) (BA4/3)	±34	−32	58	−3.6
Insula (BA13)	±42	−10	−4	−2.7
Prefrontal cortex (PFC) (BA9)	±24	64	−2	−2.83

* STN StimNet was a hemispherically derived network therefore regions are symmetrically represented on the left or right

a Subthalamic complex consists of the subthalamic nucleus and ventrally located substantia nigra

b Supplementary motor area complex consists of the SMA and pre-SMA

## Data Availability

Deidentified data will be made available on reasonable request from interested investigators for the purpose of replicating results.
